# Beyond Boundaries: The Role of Artificial Intelligence in Shaping the Future Careers of Medical Students in Saudi Arabia

**DOI:** 10.7759/cureus.69332

**Published:** 2024-09-13

**Authors:** Dalia M Alammari, Rola E Melebari, Jumanah A Alshaikh, Lara B Alotaibi, Hanan S Basabeen, Alanoud F Saleh

**Affiliations:** 1 Pathology and Immunology, Ibn Sina National College for Medical Studies, Jeddah, SAU; 2 College of Medicine, Ibn Sina National College for Medical Studies, Jeddah, SAU

**Keywords:** ai applications, ai in medical carriers, artificial intelligence, cross-sectional study, deep learning

## Abstract

Introduction: Artificial intelligence (AI) stands at the forefront of revolutionizing healthcare, wielding its computational prowess to navigate the labyrinth of medical data with unprecedented precision. In this study, we delved into the perspectives of medical students in the Kingdom of Saudi Arabia (KSA) regarding AI's seismic impact on their careers and the medical landscape.

Methods: A cross-sectional study conducted from February to December 2023 examined the impact of AI on the future of medical students’ careers in KSA, surveying approximately 400 participants, including Saudi medical students and interns, and uncovering a fascinating tapestry of perceptions.

Results: Astonishingly, 75.4% of respondents boasted familiarity with AI, heralding its transformative potential. A resounding 88.9% lauded its capacity to enrich medical education, marking a paradigm shift in learning approaches. However, amidst this wave of optimism, shadows of apprehension loomed. A staggering 42.5% harbored concerns of AI precipitating job displacement, while 34.4% envisioned a future where AI usurps traditional doctor roles. Despite this dichotomy, there existed a unanimous recognition of the symbiotic relationship between AI and human healthcare professionals, heralding an era of collaborative synergy.

Conclusion: Our findings underscored a critical need for educational initiatives to assuage fears and facilitate the seamless integration of AI into clinical practice. Moreover, AI's burgeoning influence in diagnostic radiology and personalized healthcare plans emerged as catalysts propelling the domain of precision medicine into uncharted realms of innovation. As AI reshapes the contours of healthcare delivery, it not only promises unparalleled efficiency but also holds the key to unlocking new frontiers in treatment outcomes and accessibility, heralding a transformative epoch in the annals of medicine.

## Introduction

Artificial intelligence (AI) represents a revolutionary paradigm in modern technology, encompassing the science of developing machines capable of simulating human intelligence across various domains, with medicine being a prominent frontier. Relentless progress in computing power and the advent of sophisticated cloud-based data systems, notably within machine learning and deep learning (DL), have propelled AI to the forefront of innovation in clinical medicine and medical research. These advancements have unlocked many possibilities, empowering AI to decipher complex medical data, facilitate precise diagnoses, and even forecast patient outcomes with remarkable accuracy. This convergence of cutting-edge technology with healthcare not only promises to revolutionize medical practice but also holds the potential to redefine our understanding of disease mechanisms and treatment strategies. As such, the integration of AI into the fabric of healthcare represents a pivotal milestone in our quest for enhanced patient care and medical breakthroughs [1-3].

AI has permeated deeply into the realm of medical sciences, heralding a new era of innovation and efficiency. Its applications span a wide spectrum, from the precise diagnosis of patients to the intricate process of drug discovery and development. In clinical settings, AI acts as a bridge, enhancing communication between physicians and patients, streamlining the transcription of critical medical documents such as prescriptions, and even enabling remote treatment interventions. Furthermore, AI stands poised to revolutionize surgical pathology, promising rapid and accurate results that not only expedite patient care but also empower pathologists to focus on higher-level diagnostic and consultative tasks. By seamlessly integrating molecular, morphologic, and clinical information, AI facilitates the discernment of complex cases, aiding in determining prognosis with objective precision. While historically, computer systems have often surpassed human capabilities in task execution, recent advancements in AI have elevated algorithms to levels of accuracy on par with human experts, marking a significant milestone in the convergence of technology and medicine. As AI continues to evolve, its potential to reshape the landscape of the medical sciences grows exponentially, promising transformative advancements and improved patient outcomes on a global scale [4-6].

The success of Google's AlphaGo program in 2016 advanced DL, bringing AI into a new era and spurring interest in developing and implementing AI systems in many fields, including healthcare. Between 1997 and 2015, fewer than 30 AI medical devices were approved by the U.S. Food and Drug Administration (FDA), but by mid-2021, this number had increased to more than 350 [1]. Furthermore, a growing number of studies have shown that DL algorithms are at least as good as physicians' algorithms in terms of diagnostic performance [5-7]. This means that DL-driven AI has the potential to provide a range of benefits in clinical care. For example, DL-driven AI can be used to solve current problems such as workforce shortages and provide consistency by reducing variability in medical practice and standardizing the quality of care [4,8]. Some believe that the increasing use of AI will fundamentally change the nature of health care and clinical practice [9]. However, this gradual shift may also cause concern in the medical community, as the introduction of new technologies requires changes in medical practice [7].

According to a survey conducted in Saudi Arabia on the factors influencing medical students' decisions regarding radiology as a career, high salaries, fewer hours per week, and job flexibility were the main motivators for respondents to choose radiology; however, the lack of direct patient contact and use of developed technology were the main deterrents [8]. In a follow-up study, the perceived advantages of AI in clinical and professional contexts were evaluated along with the degree of knowledge Saudi radiology personnel had of the technology. The degree of experience radiology staff have with AI has a big impact on how ready they are to accept, understand, and use this technology in clinical settings. The individuals exhibited a favorable perception of AI, indicating rational comprehension and a strong desire to investigate and apply it in medical contexts. However, this view can change with proper training and educational campaigns, some participants expressed worries that AI development would jeopardize their careers [9]. In addition, a survey carried out in December 2019 aimed to evaluate the perspectives of radiologists, surgeons, and medical students concerning critical elements influencing radiology's future. Turf wars, teleradiology, AI, and 3D printing were some of the important subjects covered. Concerning AI, in particular, radiologists expected improved workflow effectiveness and largely supported its applauding cation. On the other hand, there was a greater level of skepticism regarding the use of AI among medical students and surgeons. Notably among medical students, there was a perception that AI may jeopardize the job of diagnostic radiologists. This was a reflection of concerns over the disruptive potential of AI technologies to conventional medical procedures [9,10].

The purpose of this study is to shed light on the differences in healthcare students' knowledge and perspectives on AI in various academic fields. It also aims to emphasize their concerns about the use of AI in healthcare settings. By doing this, the study aims to pinpoint and fill in the knowledge gaps on AI technology that are common among Saudi medical students, advancing a more thorough comprehension of its applications in the healthcare industry.

## Materials and methods

A cross-sectional study conducted from February to December 2023 examined the impact of AI on the future of medical students' careers in the Kingdom of Saudi Arabia (KSA). Employing a convenient non-probability sampling technique, the study aimed to capture perspectives from approximately 400 participants, determined using the single population formula in Epi Info (Centers for Disease Control and Prevention (CDC), Atlanta, Georgia, USA), with a 95% confidence level and a 5% margin of error. The protocol had been approved (protocol identification: 040MP/CR28032023) by the Institutional Research Review Board of Ibn Sina National College-Research and Innovation Center at Ibn Sina National College for Medical Studies.

Participant selection criteria

Inclusion Criteria

The study included Saudi male and female students enrolled in medical programs, specifically targeting those in their fourth, fifth, and sixth years, as well as intern doctors. These groups were chosen to ensure a comprehensive understanding of the perspectives of medical students at different stages of their education and training.

Exclusion Criteria

Non-Saudi medical students were excluded from participation in the study. Additionally, individuals who incorrectly completed their questionnaires were excluded from the analysis to maintain the integrity and reliability of the data.

Data collection method

Utilizing a web-based platform, an online survey was developed and distributed to gather insights into the correlation between AI and the future career prospects of medical students in the KSA. The survey comprised two main sections: the first segment captured demographic information such as gender, nationality, and academic year, while the second section consisted of 14 carefully curated questions exploring participants' perceptions of AI's potential impact on the medical field (Table 5 in Appendices). Prior to commencing the survey, all respondents were assured of the confidentiality of their responses and informed that their data would be used solely for research purposes. Additionally, participants were explicitly informed of their right to withdraw from the study at any point.

Data analysis and interpretation

The analysis utilized the Statistical Package for the Social Sciences (IBM SPSS Statistics for Windows, IBM Corp., Version 22.0, Armonk, NY) software for a thorough examination of the data. Descriptive statistics, such as frequencies, pie charts, and bar charts, were employed to characterize categorical variables such as gender and academic year. Additionally, to assess the impact of AI on future unemployment rates, stratified by gender and medical student status, a standard Chi-square test was conducted. A significance level of p < 0.05, with a confidence level of 95%, was used to determine statistical significance. This analysis aims to offer a nuanced understanding of medical students' viewpoints regarding the potential impact of AI on their careers and the broader healthcare field.

## Results

Demographic examination of medical students exploring diverse perspectives on AI’s impact on job security

Table 1 concluded that among female participants, 32.4% (n=67) expressed concern about potential job loss due to AI, while 26.1% (n=54) disagreed, and 17.9% (n=37) were uncertain. Conversely, among males, 10.1% (n=21) believed in job loss, 6.8% (n=14) disagreed, and 6.8% (n=14) were uncertain. The calculated p-value of 0.683 which is more than 0.05 suggests that there is no significant relationship between gender and perceptions of AI-induced job loss.

**Table 1:** Unveiling Medical Students' Views: AI's Influence on Career Prospects and Its Revolutionary Impact on Medicine. * P < 0.05, N=207 and the results have been represented as percentages (%).

Variable		Does the application of artificial intelligence (AI) lead to a risk of job loss?						Total		P-value*	Chi-square
		No		Yes		I don’t know					
		No.	%	No.	%	No.	%	No.	%		34.235
Fears about using AI among medical students	No	19	9.2	36	17.4	13	6.3	68	32.9	0.017	
	Yes	7	3.4	71	34.3	10	4.8	88	42.5		
	I don’t know	5	2.4	24	11.6	22	10.6	51	24.6		
Total		31	15	131	63.3	45	21.7	207	100		
AI devalues the medical profession	No	64	30.9	39	18.8	20	9.7	123	59.4	0.004	89.123
	Yes	3	1.4	43	20.8	10	4.8	56	27.1		
	I don’t know	1	0.5	6	2.9	21	10.1	28	13.5		
Total		68	32.9	88	42.5	51	24.6	207	100		
AI going to diminish the importance of the role of doctors in the future	No	58	28	21	10.1	13	6.3	92	44.4	0.022	111.647
	Yes	6	2.9	55	26.6	10	4.8	71	34.4		
	I don’t know	4	1.9	12	5.8	28	13.5	44	21.3		
Total		68	32.9	88	42.5	51	24.6	207	100		
AI can replace doctors’ role in the future	No	60	29	40	19.3	22	10.6	122	58.9	0.004	108.627
	yes	5	2.4	46	22.2	4	1.9	55	26.6		
	I don’t know	3	1.4	2	1	25	12.1	30	14.5		
Total		68	32.9	88	42.5	51	24.6	207	100		
AI will increase the unemployment rate in the future	No	42	20.3	12	5.8	7	3.4	61	29.5	0.03	116.373
	Yes	12	5.8	70	33.8	12	5.8	94	45.4		
	I don’t know	14	6.8	6	2.9	32	15.5	52	25.1		
Total		68	32.9	88	42.5	51	24.6	207	100		

In the medical student cohort, 18.8% (n=39) of sixth-year students expressed belief in job loss, with a p-value of 0.007 which is more than 0.05 indicating no significant correlation. Notably, fifth-year students exhibited the lowest level of concern at 3.9% (n=8). Regarding nationality, 31.9% (n=66) of Saudi participants perceived job loss, compared to 10.6% (n=22) of non-Saudis. The calculated p-value of 0.603 which is more than 0.05 suggests that there is no substantial link between nationality and perceptions of AI-induced job loss.

Assessing participants' familiarity and depth of understanding the AI: Implications for career concerns among medical students

The findings in Table 2 indicate that a significant majority (75.4%, n=156) of participants possess a background in AI, suggesting a substantial familiarity with the subject matter. Conversely, 16.4% (n=34) acknowledged having no prior exposure to AI, while 8.2% (n=17) expressed uncertainty regarding their knowledge. The statistical analysis showed a p-value of 0.763, which is more than 0.05, revealing no significant association between participants' AI background and concerns about future job security.

**Table 2:** Perceptions of Job Loss Risk Due to Artificial Intelligence Among Medical Students: A Demographic Analysis. * P < 0.05, N=207 and the results have been represented as percentages (%).

Variable		Does the application of artificial intelligence (AI) lead to a risk of job loss?						Total		P-value*	Chi-square
		Yes		No		I don’t know					
		No. of subj	%	No.	%	No.	%	No.	%		
Gender	Male	21	10.1%	14	6.8%	14	6.8%	49	23.7%	0.683	0.763
	Female	67	32.4%	54	26.1%	37	17.9%	158	76.3%		
Total		88	42.5%	68	32.9%	51	24.6%	207	100%		
Academic year	Fourth	31	15%	20	9.7%	11	5.3%	62	30%	0.007	17.624
	Fifth	8	3.9%	0	0%	9	4.3%	17	8.2%		
	Sixth	39	18.8%	34	16.4%	19	9.2%	92	44.4%		
	Intern	10	4.9%	14	6.8%	12	5.8%	36	17.4%		
Total		88	42.5%	68	32.9%	51	24.6%	207	100%		
Nationality	Saudi	66	31.9%	53	25.6%	42	20.3%	161	77.8%	0.603	1.012
	Non-Saudi	22	10.6%	15	7.2%	9	4.3%	46	22.2%		
Total		88	42.5%	68	32.9%	51	24.6%	207	100%		

Regarding the depth of understanding, 24.2% (n=50) claimed to have a deep understanding of AI, while the majority 60.4% (n= 125) reported lacking such depth. Notably, 15.5% (n= 32) of participants remained uncertain about their level of understanding. The statistical analysis showed a p-value of 0.745, which is more than 0.05, suggesting that there is no significant association between the depth of participants' understanding of AI and concerns about future job security.

Perceptions and concerns regarding AI’s role in medical education and practice

Table 3 represents that the vast majority of respondents (88.9%, n=184) acknowledged the benefits of integrating AI into medical education, while a minority 3.9% (n=8) expressed skepticism, and a smaller fraction (7.2%, n=7.2) reported uncertainty. The statistical analysis showed a p-value of 0.017, which is less than 0.05, unveiling a notable correlation between participants who recognized AI's advantages for medical students and apprehensions about potential job displacement in the future.

**Table 3:** Exploring Medical Students' Perceptions of the Potential Impact of Artificial Intelligence on Career Trajectories. * P > 0.05, N=207 and the results have been represented as percentages (%).

Variable		Does the application of artificial intelligence (AI) lead to a risk of job loss?						Total		P-value*	Chi-square
		No		Yes		I don’t know					
		No.	%	No.	%	No.	%	No.	%		
Background in AI?	No	8	3.9%	16	7.7%	10	4.8%	34	16.4%	0.763	15.202
	Yes	55	26.6%	70	33.8%	31	15.0%	156	75.4%		
	I don’t know	5	2.4%	2	1.0%	10	4.8%	17	8.2%		
Total		68	32.9%	88	42.5%	51	24.6%	207	100%		
Deep understanding of AI?	No	41	19.8%	53	25.6%	31	15.0%	125	60.4%	0.745	4.789
	Yes	13	6.3%	26	12.6%	11	5.3%	50	24.2%		
	I don’t know	14	6.8%	9	4.3%	9	4.3%	32	15.5%		
Total		68	32.9%	88	42.5%	51	24.6%	207	100%		
Medical students should learn about AI?	No	12	5.8%	13	6.3%	1	0.5%	26	12.6%	0.10	12.453
	Yes	48	23.2%	69	33.3%	39	18.8%	156	75.4%		
	I don’t know	8	3.9%	6	2.9%	11	5.3%	25	12.1%		
Total		68	32.9%	88	42.5%	51	24.6%	207	100%		

Regarding specific applications, a significant proportion believed AI is valuable in aiding diagnosis (74.9%, n=155) and facilitating decision-making (72.9%, n=151). The statistical analyses for these perceptions (p = 0.854, p = 0.205, respectively) which is more than 0.05 indicated no significant association between participants' views on AI's role in diagnosis and decision-making and concerns about future job security. However, a noteworthy (71%, n=147) of participants believed AI could reduce medical errors, with a statistical analysis of 0.022, which is less than 0.05, revealing a significant correlation between this belief and apprehensions about future job loss.

Examining concerns surrounding AI integration in medicine

Participants in Table 4 expressed a range of concerns regarding AI integration, with 42.5% (n=88) reporting fears, 32.9% (n=68) indicating no fears, and 24.6% (n=24.6) remaining uncertain. The statistical analysis showed a p-value of 0.017, which is less than 0.05, unveiling a significant correlation between participants' familiarity with AI and their apprehensions about its use in medical education.

**Table 4:** How Artificial Intelligence Can Contribute to Medical Services. * P < 0.05, N=207 and the results have been represented as percentages (%).

Variable		No		Yes		I don’t know				P-value*	Chi-square
		No.	%	No.	%	No.	%	No.	%		
Artificial intelligence is beneficial for medical students	No	2	1.0%	5	2.4%	1	0.5%	8	3.9%	0.017	12.250
	Yes	64	30.9%	97	38.2%	41	19.8%	184	88.9%		
	I don’t know	2	1%	4	1.9%	9	4.3%	15	7.2%		
Total		68	32.9%	88	42.5%	51	24.6%	207	100%		
Artificial intelligence helpful using in diagnosis	No	7	3.4%	15	7.2%	4	1.9%	26	12.6%	0.854	10.765
	Yes	49	23.7%	69	33.3%	37	17.9%	155	74.9%		
	I don’t know	12	5.8%	4	1.9%	10	4.8%	26	12.6%		
Total		68	32.9%	88	42.5%	51	24.6%	207	100%		
Artificial intelligence can reduce medical errors.	No	10	4.8%	11	5.3%	1	0.5%	22	10.6%	0.022	10.363
	Yes	46	22.2%	66	31.9%	35	16.9%	147	71%		
	I don’t know	12	5.8%	11	5.3%	15	7.2%	38	18.4%		
Total		68	32.9%	88	42.5%	51	24.6%	207	100%		
Artificial intelligence helpful in making decisions	No	11	5.3%	11	5.3%	5	2.4%	27	13%	0.205	2.765
	Yes	48	23.2%	67	32.4%	36	17.4%	151	72.9%		
	I don’t know	9	4.3%	10	4.8%	10	4.8%	29	14%		
Total		68	32.9%	88	42.5%	51	24.6%	207	100%		

Likewise, significant associations emerged in participants' perceptions of AI potentially devaluing the medical profession (p = 0.04), diminishing the significance of doctors' roles in the future (p = 0.022), AI replacing doctors' roles in the future (p = 0.04), and AI contributing to future unemployment (p = 0.03), which all are less than 0.05. These findings underscore the profound impact of participants' AI background on their perceptions and concerns regarding AI's integration into the medical domain.

## Discussion

The overarching objective of this research is to furnish a nuanced and scientifically sound understanding of the comprehension and viewpoints of healthcare students in Saudi Arabia concerning the influence of AI on their forthcoming professional trajectories. The resultant findings are poised to illuminate prevalent concerns, potential challenges, and anticipated developments within this domain. Moreover, these insights are anticipated to foster informed discourse and strategic planning on the seamless integration of AI within medical education and clinical practice, thereby catalyzing transformative advancements in healthcare delivery and education within the Saudi context.

The provided data offers insights into the perceptions of individuals, particularly delineated by gender and educational background (medical students), concerning the potential ramifications of AI on job security. Notably, a higher percentage of females (32.4%) expressed apprehensions regarding AI-induced job loss compared to their male counterparts (10.1%), with a notable proportion of both genders exhibiting uncertainty (17.9% for females and 6.8% for males). However, the statistical analysis revealed p-values exceeding 0.05, indicating a lack of significant statistical relationship between gender and these perceptions.

In the context of medical students, distinct trends emerge across academic years. Sixth-year students displayed the highest level of concern regarding AI-induced job loss (18.8%), followed by fourth-year students (15%), while fifth-year students exhibited the lowest level of concern at 3.9%. Similarly, a notable percentage of medical students across different years expressed uncertainty on this matter. Consistent with the gender analysis, the associated p-values for these findings also surpassed 0.05, suggesting no significant statistical relationship between the year of study and these perceptions.

A comprehensive discussion of these results can be augmented by referencing existing research articles. Notably, various studies have delved into the multifaceted impact of AI on employment perceptions. For instance, Perifanis and Kitsios elucidated the heterogeneous nature of public perceptions concerning AI's influence on job security, highlighting its susceptibility to factors such as gender, educational attainment, and occupational background. It is paramount to acknowledge the dynamism inherent in the AI landscape, underscoring that these perceptions are subject to evolution over time in tandem with technological advancements and heightened awareness among individuals about AI's potential implications [11].

Another study discussed how educational background and awareness of AI technologies can influence perceptions of job security. They found that individuals with a higher level of AI-related education tend to be more optimistic about AI's potential to create new job opportunities rather than displacing existing ones [12]. The analysis indicates a mixed relationship between people's beliefs about AI and their concerns about job loss. It is essential to consider these findings in the broader context of existing research on this topic.

Additionally, a notable proportion of Saudi participants (31.9%) harbor concerns regarding the potential risk of job loss attributable to AI. However, the associated p-value (0.745) exceeds the threshold of 0.05, suggesting the absence of a statistically significant relationship between these apprehensions and participants' background in AI. This finding corroborates previous research, such as that which underscores the diverse nature of public perceptions concerning AI's impact on employment. Such perceptions, it appears, are not exclusively contingent upon individuals' familiarity with the technology.

The findings from this study reveal a significant correlation between medical students' awareness of the advantages of AI in healthcare and their apprehensions regarding future job security. With a p-value of 0.017, indicative of a strong association, the null hypothesis is rejected. This outcome suggests that individuals who possess greater knowledge about the positive impacts of AI in healthcare are more inclined to harbor concerns about potential job displacement. Interestingly, these results diverge from those reported by Pinto Dos Santos et al. [13], whose study found that medical students do not express significant worries about AI replacing human radiologists, and are cognizant of the potential applications and ramifications of AI in radiology and medicine. Such disparate findings underscore the intricate and multifaceted nature of medical students' attitudes toward AI in healthcare. Moreover, they highlight the potential influence of education and exposure in shaping students' perceptions and mitigating their apprehensions. Additionally, the swift evolution of AI technologies underscores the need for ongoing research to comprehensively understand and address the evolving dynamics of medical students' perceptions and concerns in this domain.

Moreover, the analysis of the association between medical students' awareness of AI's capacity to mitigate medical errors and their concerns regarding job security also bolsters the hypothesis, as evidenced by a p-value of 0.002. This finding aligns with the conclusions drawn by Ardon and Schmidt in 2020, who underscored that the perception of AI as a mechanism for enhancing patient safety exacerbates apprehensions about job stability among medical professionals. The outcomes of this study underscore a significant correlation between the integration of AI and the perceived risk of job displacement among medical students, alongside their apprehensions surrounding AI adoption [14]. This outcome lends credence to the rejection of the null hypothesis, signifying a tangible relationship between these variables. Numerous scholarly articles have delved into the ramifications of AI in medical practice, yielding both supportive and contradictory evidence on this subject. This discourse will scrutinize the diverse viewpoints and findings from these investigations.

One research article in 2021, titled "Artificial Intelligence in Healthcare: Transforming the Practice of Medicine," focused on the benefits of AI in medical care and showed that AI can enhance diagnostic accuracy, improve treatment planning, and reduce medical errors (p = 0.000) [15]. It argued that AI serves as a valuable tool for healthcare professionals, strengthening their decision-making process and providing them with additional insights. This perspective suggests that the implementation of AI in medical care can ultimately improve patient outcomes and enhance the role of doctors as they leverage AI technology [14].

Contrarily, a study conducted by Richardson et al., titled "Patient Apprehensions About the Use of Artificial Intelligence in Healthcare," delved into the apprehensions surrounding AI in healthcare, encompassing concerns about potential job displacement and the perceived devaluation of the medical profession (p = 0.000). The findings substantiated the notion that AI applications could precipitate job loss risk. Moreover, respondents articulated concerns about AI potentially diminishing the significance of doctors' roles in the future, with apprehensions extending to the possibility of AI ultimately replacing them. These results resonate with the sentiments echoed among medical students in the present study [16].

Additionally, an investigation by Ahuja in 2019 scrutinized the ramifications of AI on future employment rates within the medical student cohort (p = 0.000). The study unearthed that AI integration in healthcare could potentially elevate the unemployment rate by automating certain routine tasks, thereby diminishing the demand for human labor in specific domains [17]. These findings mirror the apprehensions voiced by medical students in the current study.

Conversely, a study in 2023 by Sezgin provided a contrasting perspective, positing that AI has the potential to augment, rather than supplant, the role of doctors. The research demonstrated that AI integration in healthcare can bolster efficiency, enabling physicians to concentrate on intricate patient care and personalized medicine (p = 0.000). Emphasizing the indispensability of the human element in the doctor-patient relationship, the study advocated for perceiving AI as a complementary tool rather than a disruptive force [18].

Limitations of the study

While the research examining the impact of AI on the careers of medical students in Saudi Arabia provides valuable insights, several limitations must be acknowledged. Firstly, the study's sample size is small and may not adequately represent the entire population. Additionally, the cross-sectional approach utilized restricts the study's ability to provide longitudinal insights into the evolving perceptions of medical students over time. Moreover, reliance on self-reported data introduces potential biases and inaccuracies in the findings. The survey instrument employed may not capture the full complexity of participants' attitudes and experiences regarding AI in healthcare. Furthermore, the study's limited timeframe fails to account for the rapid advancements and evolving nature of AI technologies. The undisclosed response rate further complicates the interpretation of the results. Additionally, the focus solely on Saudi Arabia limits the generalizability of the findings to other cultural and regional contexts. Lastly, the study's reliance on p-values to determine significance may introduce biases and overlook other important factors influencing the outcomes. These limitations warrant a cautious interpretation of the study findings and underscore the need for further research to address these gaps and enhance the understanding of AI's impact on medical careers.

## Conclusions

In conclusion, this research delved into the multifaceted perceptions and concerns surrounding the integration of AI in healthcare, particularly among medical students and intern doctors in Saudi Arabia. Through a comprehensive analysis of survey data and a review of relevant literature, several key findings emerged. Firstly, the study elucidated a significant level of apprehension among participants regarding the potential impact of AI on job security within the medical profession. Both genders expressed varying degrees of concern, with females exhibiting a higher level of apprehension compared to males. Furthermore, while different academic years showed differing levels of concern, uncertainty about AI-induced job loss was prevalent across all cohorts. The analysis also revealed complex relationships between participants' awareness of AI's benefits in healthcare and their fears of job displacement. Interestingly, individuals who acknowledged AI's potential to reduce medical errors were more likely to harbor concerns about future job security. These findings highlight the nuanced attitudes of medical students toward AI and underscore the importance of education and exposure in shaping perceptions.

The discussion also contextualized the study findings within the broader landscape of existing research. Various studies have presented divergent perspectives on AI's impact on healthcare employment, with some emphasizing its potential to augment medical practice and others raising concerns about job displacement. These differing viewpoints underscore the need for ongoing dialogue and research to navigate the evolving intersection of AI and healthcare. Building on these insights, it is evident that effective education and collaboration are crucial in addressing concerns and facilitating the successful integration of AI into medical practice. By fostering a deeper understanding of AI's capabilities and limitations, healthcare professionals can harness its potential to enhance patient care while mitigating anxieties about job security. In essence, this study contributes valuable insights into the complex interplay between AI, medical education, and professional practice. By shedding light on the perceptions and concerns of medical students and intern doctors, it lays a foundation for future research and initiatives aimed at maximizing the benefits of AI while safeguarding the integrity of the medical profession.

## Appendices

**Table 5:** Questionnaire

Questionnaire			
Section 1: Sociodemographic data			
Gender	Male		Female
Academic year	4^th^, 5^th^ or 6^th^ year medical student		Intern
Nationality	Saudi		non-Saudi
Section 2: All questions are close-ended (Likert Question)			
1-Do you have a background in AI?	Yes	No	I do not know
2-In your opinion, do you have a deep understanding of AI?	Yes	No	I do not know
3-Do you think medical students should learn about AI?	Yes	No	I do not know
4-Do you think using AI has benefits for medical students?	Yes	No	I do not know
5-Do you think AI can be used or helped with the diagnosis of diseases?	Yes	No	I do not know
6-Is there a relationship between using AI and reducing medical errors?	Yes	No	I do not know
7-Do you believe AI is useful in helping physicians make decisions?	Yes	No	I do not know
8-Do you think using AI might help a physician make more accurate decisions?	Yes	No	I do not know
9-Are there any fears about using AI among medical students?	Yes	No	I do not know
10-Does AI devalue the medical profession?	Yes	No	I do not know
11-Is AI going to diminish the importance of the role of doctors in the future?	Yes	No	I do not know
12-Does the application of AI lead to a risk of job loss?	Yes	No	I do not know
13-Do you think AI can replace doctors' roles in the future?	Yes	No	I do not know
14-As a medical student, do you think AI will increase the unemployment rate in the future?	Yes	No	I do not know

## References

[1] Chen M, Zhang B, Cai Z (2022). Acceptance of clinical artificial intelligence among physicians and medical students: a systematic review with cross-sectional survey. Front Med (Lausanne).

[2] Bhattad PB, Jain V (2020). Artificial intelligence in modern medicine - the evolving necessity of the present and role in transforming the future of medical care. Cureus.

[3] Alowais SA, Alghamdi SS, Alsuhebany N (2023). Revolutionizing healthcare: the role of artificial intelligence in clinical practice. BMC Med Educ.

[4] Najjar R (2023). Redefining radiology: a review of artificial intelligence integration in medical imaging. Diagnostics (Basel).

[5] Basu K, Sinha R, Ong A, Basu T (2020). Artificial intelligence: How is it changing medical sciences and its future?. Indian J Dermatol.

[6] Ahmad Z, Rahim S, Zubair M, Abdul-Ghafar J (2021). Artificial intelligence (AI) in medicine, current applications and future role with special emphasis on its potential and promise in pathology: present and future impact, obstacles including costs and acceptance among pathologists, practical and philosophical considerations. A comprehensive review. Diagn Pathol.

[7] Abduljabbar AH, Alnajjar SF, Alshamrani H, Bashamakh LF, Alshehri HZ, Alqulayti WM, Wazzan MA (2020). The influence of gender on the choice of radiology as a specialty among medical students in Saudi Arabia: cross-sectional study. Interact J Med Res.

[8] Qurashi AA, Alanazi RK, Alhazmi YM, Almohammadi AS, Alsharif WM, Alshamrani KM (2021). Saudi radiology personnel’s perceptions of artificial intelligence implementation: a cross-sectional study. J Multidiscip Healthc.

[9] van Hoek J, Huber A, Leichtle A (2019). A survey on the future of radiology among radiologists, medical students and surgeons: students and surgeons tend to be more skeptical about artificial intelligence and radiologists may fear that other disciplines take over. Eur J Radiol.

[10] Huisman M, Ranschaert E, Parker W (2021). An international survey on AI in radiology in 1,041 radiologists and radiology residents part 1: fear of replacement, knowledge, and attitude. Eur Radiol.

[11] Perifanis NA, Kitsios F (2023). Investigating the influence of artificial intelligence on business value in the digital era of strategy: a literature review. Information.

[12] Seo K, Tang J, Roll I, Fels S, Yoon D (2021). The impact of artificial intelligence on learner-instructor interaction in online learning. Int J Educ Technol High Educ.

[13] Pinto Dos Santos D, Giese D, Brodehl S (2019). Medical students' attitude towards artificial intelligence: a multicentre survey. Eur Radiol.

[14] Ardon O, Schmidt RL (2020). Clinical Laboratory employees’ attitudes toward artificial intelligence. Lab Med.

[15] Bajwa J, Munir U, Nori A, Williams B (2021). Artificial intelligence in healthcare: transforming the practice of medicine. Future Healthc J.

[16] Richardson JP, Smith C, Curtis S, Watson S, Zhu X, Barry B, Sharp RR (2021). Patient apprehensions about the use of artificial intelligence in healthcare. NPJ Digit Med.

[17] Ahuja AS (2019). The impact of artificial intelligence in medicine on the future role of the physician. PeerJ.

[18] Sezgin E (2023). Artificial intelligence in healthcare: complementing, not replacing, doctors and healthcare providers. Digit Health.

